# Lowering light intensity while extending photoperiod at a constant daily light integral synergistically interacts with warm temperature to enhance leaf expansion and crop yield in lettuce in the absence of far-red light

**DOI:** 10.3389/fpls.2025.1529455

**Published:** 2025-01-24

**Authors:** Sang Jun Jeong, Shuyang Zhen, Qianwen Zhang, Genhua Niu

**Affiliations:** ^1^ Department of Horticultural Sciences, Texas A&M University, College Station, TX, United States; ^2^ Texas A&M AgriLife Research, Dallas, TX, United States; ^3^ Truck Crops Branch Experiment Station, Mississippi State University, Crystal Springs, MS, United States

**Keywords:** indoor farming, photon capture, phytochrome photoequilibrium, plant yield, antioxidant capacity

## Abstract

**Introduction:**

Low light intensity and far-red (FR) light act as shade signals to induce specific morphological changes mediated by plant photoreceptors phytochromes (PHYs). Applying FR light or lowering light intensity over a longer photoperiod at a constant daily light integral (DLI) can increase crop yield by enhancing leaf expansion and photon capture. However, PHY activity is also dependent on temperature. We aimed to investigate the interactive effects of FR light, light intensity, photoperiod, and temperature on plant growth and morphology.

**Methods:**

Lettuce (*Lactuca sativa* L.) ‘Rex’ was grown under three temperatures (20, 24, and 28 °C), each containing six light treatments [two levels of FR light (0 and 20% FR in total photon flux density from 400-800 nm) x three light intensities (150, 200, and 300 μmol m^-2^ s^-1^)]. As light intensity increased, photoperiod was reduced (150, 200, and 300 μmol m^-2^ s^-1^ with photoperiods of 24 h, 18 h, and 12 h, respectively) to maintain a constant DLI of 13 mol m^-2^ d^-1^.

**Results:**

Under 0% FR light, the combination of lower light intensity/longer photoperiod and warmer temperature synergistically enhanced leaf expansion and photon capture; however, this interactive effect disappeared under 20% FR light. Stem elongation exhibited an opposite response pattern to leaf expansion; lower light intensity and warm temperature had a synergistic enhancement on stem elongation under 20% FR light, but not under 0% FR light. Shoot dry weight responded to the light and temperature factors similarly to total leaf area. Our results showed that plant biomass accumulation depended primarily on photon capture (r^2^ = 0.93), rather than single-leaf photosynthetic efficiency. Antioxidant capacity was generally reduced by lower light intensity and FR light, but the reduction could be compensated by warmer temperatures.

**Discussion:**

Thus, we concluded that applying lower light intensity over a longer photoperiod, combined with warm temperature, can effectively maximize leaf expansion and crop yield while maintaining nutritional quality in the absence of FR light. However, under strong shade signals composed of FR light, low light intensity, and warm temperature, lettuce prioritizes stem elongation at the expense of leaf expansion, leading to reduced crop yield.

## 1 Introduction

Indoor farming has emerged as a viable alternative to traditional agriculture, providing precisely controlled environments that mitigate the challenges posed by unpredictable weather and extreme conditions. However, due to high production costs in indoor farms, there is a rising demand to optimize environmental factors to enhance crop yield while maximizing resource use efficiency. In indoor farming practices, far-red (FR; 700-800 nm) light, a common shade signal, has been strategically utilized to increase crop yield by altering plant morphology, particularly by enhancing leaf expansion (Park and Runkle, 2017; Meng and Runkle, 2019; Legendre and van Iersel, 2021). The morphological response to FR light is primarily mediated by phytochromes (PHYs), a family of photoreceptors. Specifically, FR light can convert the active PHYs into the inactive form, leading to the accumulation of growth-promoting hormones such as auxin and gibberellins (Bou-Torrent et al., 2014; de Wit et al., 2016; Yang and Li, 2017; Fernández-Milmanda and Ballaré, 2021). Similar to FR light, lower light intensity, another shade signal, can also induce morphological responses by decreasing the activity of PHYs and stimulating hormonal changes (Vandenbussche et al., 2003; Jiang et al., 2021; Brini et al., 2022). Previous research has reported that reducing light intensity while extending photoperiod at the same daily light integral (DLI) promoted leaf expansion and plant growth in diverse crops, including lettuce, mizuna, spinach, beet, radish, cabbage, tomato, and rudbeckia (Soffe et al., 1977; Velez-Ramirez et al., 2014; Weaver and van Iersel, 2020; Palmer and van Iersel, 2020; Elkins and van Iersel, 2020a; Meng and Severin, 2024).

The accelerated growth under FR light and a lower light intensity/longer photoperiod at the same DLI were attributed to not only morphological differences but also improved photochemical efficiency. For example, adding FR light to a background of shorter-wavelength light has been shown to improve quantum yield of PSII as well as the CO_2_ assimilation rate at both the leaf and plant canopy levels in short-term photosynthesis studies (Zhen and van Iersel, 2017; Zhen and Bugbee, 2020a). Similarly, applying a lower intensity light over longer photoperiod also resulted in higher photochemical efficiency (Elkins and van Iersel, 2020b). However, prolonged exposure to FR light and lower light intensity led to a significant decrease in the single-leaf net CO_2_ assimilation rate, likely due to morphological and physiological acclimation to shade, such as reductions in leaf thickness and photosynthetic pigment contents (Zou et al., 2019; Palmer and van Iersel, 2020). Nevertheless, at canopy level, plant biomass accumulation may increase under long-term acclimation to FR light due to the enhanced leaf expansion and photon capture (Zhen and Bugbee, 2020b).

However, as the shade signals (i.e., FR light and low light intensity) intensify, plants tend to exhibit excessive stem growth, leading to a reduction in leaf growth and ultimately reduced crop yield (Holmes and Smith, 1975; Frankland and Letendre, 1978; Robson et al., 1993; Devlin et al., 1999; Kusuma and Bugbee, 2023). Furthermore, the morphological responses to FR light and low light intensity are further affected by temperature as the steady state of PHYs, especially PHYB, is highly sensitive to temperature (Klose et al., 2015; Legris et al., 2016; Jung et al., 2016). For example, FR light and warm temperature synergistically promoted stem/hypocotyl elongation, while reducing leaf expansion and overall plant growth, in various crop species, including Arabidopsis, lettuce, kale, petunia, tomato, African marigold, and zinnia (Patel et al., 2013; Burko et al., 2022; Jeong et al., 2024a and b). Similarly, the effect of low light intensity on hypocotyl elongation also became greater as temperature increased (Legris et al., 2016 and 2017). However, the combination of low light intensity and warmer temperature (30°C) caused greater reductions in leaf expansion and plant biomass of lettuce compared to cooler temperatures (15 and 23°C) (Zhou et al., 2019). The enhancement of stem elongation induced by the shade signals under warmer temperature may be an adaptative strategy to compensate for the higher respiration demands by enabling plants to better reach unfiltered light (Romero-Montepaone et al., 2021). These findings highlight the importance of co-optimizing temperature, FR light, and light intensity to prevent excessive stem growth, which can cause a reduction in leaf expansion and overall plant growth.

Besides enhancing crop yield, manipulating environmental conditions in indoor farming offers a pathway to increase the concentration of health-promoting nutritional compounds (Thoma et al., 2020; Wong et al., 2020; Ampim et al., 2022). Previous studies have reported that while FR light and low light intensity promote leaf expansion, they often lead to a decrease in the accumulation of beneficial compounds such as chlorophyll, carotenoids, anthocyanin, phenolics, and flavonoids (Lefsrud et al., 2008; Li and Kubota, 2009; Stutte et al., 2009; Oh et al., 2009; Samuolienė et al., 2013; Bantis et al., 2016; Pérez-López et al., 2018; Zheng et al., 2018). In contrast, warm temperatures tend to enhance the contents of phenolics, flavonoids, and carotenoids in various crops such as lettuce, wheat, and spinach (Lefsrud et al., 2005; Oh et al., 2009; Shamloo et al., 2017), although a decrease in polyphenol content was observed under warm temperature in lettuce (Boo et al., 2011). These results suggest that warm temperature may compensate for the decrease in phytochemicals observed when applying FR light and low light intensity. Furthermore, previous studies indicate that light and temperature interact to influence phytochemical content, considering that their effects on phytochemical are commonly mediated by PHY signaling (Casal et al., 1987; Huq et al., 2004; Toledo-Ortiz et al., 2010; Bianchetti et al., 2020; Pashkovskiy et al., 2022).

Applying FR light and lowering light intensity while increasing photoperiod at a constant DLI have become common approaches to enhance leaf expansion and crop yield in indoor farming. However, limited information is available about how these shade signals interact with temperature to regulate plant morphology, yield, beneficial phytochemical contents, and antioxidant capacity. Thus, this study aimed to quantify the interactive effects of FR light, light intensity, photoperiod, and temperature on lettuce morphology, physiology, yield, and nutritional quality.

## 2 Materials and methods

### 2.1 Plant materials

Lettuce (*Lactuca sativa* L.) ‘Rex’ seeds were obtained from Jonny’s Selected Seeds (Winslow, ME, USA). Three to five seeds were sown in 0.45 L plastic pots (8.8 cm x 8.8 cm x 8.9 cm; l x w x h) filled with a soilless substrate (BM6; peat-moss and perlite; Berger, Saint-Modeste, QC, Canada) and germinated in a glass greenhouse. Four days after germination, seedlings were thinned to one plant per pot based on uniformity, and then transferred to growth chambers for treatments. Plants were irrigated manually with a complete nutrient solution containing 150 mg L^-1^ N and other essential nutrients, prepared using a water-soluble fertilizer (21N-2.2P-16.6K; Peters 21-5-20; The Scotts Company, Marysville, OH, USA) throughout the experiment.

### 2.2 Light and temperature treatments

The temperature of three walk-in growth chambers (4.0 m x 2.3 m x 2.5 m; l x w x h; Growtainer; Innovative Growers Equipment, Inc., Sycamore, IL, USA) was set at 20, 24, and 28°C, respectively. Each chamber was divided into six sections (l x w x h; 70 x 70 x 70 cm) using a multilayer growth rack to accommodate six light treatments: three light intensities [total photon flux density (TPFD; 400-800 nm) of 150, 200 or 300 μmol m^-2^ s^-1^] x two FR light levels (0% or 20% of FR light in TPFD). Therefore, a total of eighteen treatments were created: three temperature regimes x six light treatments (**Table 1**). Note that lower light intensity was coupled with longer photoperiod to reach a constant DLI of 13 mol m^-2^ d^-1^ in all treatments, i.e., 150 μmol m^-2^ s^-1^ for 24 h, 200 μmol m^-2^ s^-1^ for 18 h, and 300 μmol m^-2^ s^-1^ for 12 h. The spectral treatments were created using LEDs with blue (B; peak 450 nm), green (G; peak 521 nm), red (R; peak 660 nm), and FR (peak 730 nm) LEDs (PHYTOFY^®^ RL, Osram, Munich, Germany). Photon flux density at plant height (30 cm below the LEDs) was measured at fourteen points within each treatment area using a spectroradiometer (PS100; Apogee Instruments, Logan, UT, USA) (**Table 1**). To minimize any effect derived from spatial environmental variations, plants were randomly rotated daily within each treatment. Temperature in each section of chambers was monitored every 30 seconds and recorded every 20 minutes using a type-E thermocouple and a data logger (CR1000; Campbell Scientific, Logan, UT, USA). To ensure sufficient air circulation, we installed two small air mixing fans (4.7 W; CFM-9225V-145-455; Same Sky, Lake Oswego, OR, USA) in each compartment.

**Table 1:** Temperature, photoperiod, light intensity, and light spectral characteristics of eighteen treatments [three temperature x three light intensity (or photoperiod) x two light spectra].

Temperature setpoint (^o^C)	Photo- period (h)	TPFD^z^ setpoint	%FR in TPFD^y^	Light spectrum	DLI^x^	Actual temperature (°C) ± SD^w^	Actual TPFD ± SD	Estimated PPE^v^
20	12	300 300	0 20	B_30_ + G_30_ + R_240_ B_30_ + G_30_ + R_180_ + FR_60_	13 13	19.62 ± 0.72 19.61 ± 0.61	303.3 ± 19.4 299.3 ± 15.6	0.88 0.81
	18	200 200	0 20	B_20_ + G_20_ + R_160_ B_20_ + G_20_ + R_120_ + FR_40_	13 13	19.70 ± 0.48 19.70 ± 0.64	204.4 ± 11.9 201.2 ± 12.9	0.88 0.81
	24	150 150	0 20	B_15_ + G_15_ + R_120_ B_15_ + G_15_ + R_90_ + FR_30_	13 13	19.88 ± 0.54 19.81 ± 0.56	150.2 ± 7.8 150.4 ± 7.9	0.88 0.81
24	12	300 300	0 20	B_30_ + G_30_ + R_240_ B_30_ + G_30_ + R_180_ + FR_60_	13 13	24.19 ± 0.41 24.10 ± 0.44	300.4 ± 15.7 297.9 ± 18.7	0.88 0.81
	18	200 200	0 20	B_20_ + G_20_ + R_160_ B_20_ + G_20_ + R_120_ + FR_40_	13 13	24.23 ± 0.41 23.95 ± 0.38	202.8 ± 11.8 201.4 ± 13.0	0.88 0.81
	24	150 150	0 20	B_15_ + G_15_ + R_120_ B_15_ + G_15_ + R_90_ + FR_30_	13 13	24.06 ± 0.33 23.93 ± 0.29	150.0 ± 10.3 147.4 ± 9.1	0.88 0.81
28	12	300 300	0 20	B_30_ + G_30_ + R_240_ B_30_ + G_30_ + R_180_ + FR_60_	13 13	28.23 ± 0.63 28.35 ± 0.67	302.4 ± 16.6 298.6 ± 13.7	0.88 0.81
	18	200 200	0 20	B_20_ + G_20_ + R_160_ B_20_ + G_20_ + R_120_ + FR_40_	13 13	28.38 ± 0.59 28.16 ± 0.63	200.5 ± 15.9 198.2 ± 11.0	0.88 0.81
	24	150 150	0 20	B_15_ + G_15_ + R_120_ B_15_ + G_15_ + R_90_ + FR_30_	13 13	28.16 ± 0.48 28.15 ± 0.57	150.2 ± 8.3 149.3 ± 10.8	0.88 0.81

^z^TPFD, Total photon flux density (μmol m^-2^ s^-1^; 400 to 800 nm).

^y^%FR in TPFD, Percentage of far-red photons (700-800 nm) in total photon flux density (400 to 800 nm).

^x^DLI, Daily light integral (mol m^-2^ d^-1^; 400 to 800 nm).

^w^SD, Standard deviation.

^v^Estimated PPE, Phytochrome photoequilibrium calculated following Sager et al. (1988).

Light spectra consisted of blue (B; 400-500 nm), green (G; 500-600 nm), red (R; 600-700 nm), and far-red (FR; 700-800 nm) photons from light-emitting diodes. The subscript after each waveband indicates its photon flux density in μmol m^-2^ s^-1^.

### 2.3 Data collection and analysis

#### 2.3.1 Morphological and growth parameters

Plants were harvested after a 25-day treatment period. At harvest, total leaf number, leaf length and width of the most recently expanded leaf, and stem length were determined. Stem length was determined by measuring the distance from the root-shoot junction to shoot apex after detaching all the leaves. Total leaf area was measured using a leaf area meter (LI-3100C; LI-COR, Lincoln, NE, USA). Fresh weights (FW) of leaves, stems, and roots were recorded, and then the samples were dried in an 80°C drying oven for seven days to obtain dry weights (DW). Specific leaf area was calculated by dividing total leaf area by leaf DW.

To determine the total number of photons intercepted by each plant, top-down photos of the plants were taken every 5 days (0, 5, 10, 15, 20, and 25 days after treatment). ImageJ software (National Institutes of Health) was used to calculate the projected leaf area using the top-down photos. Based on the projected leaf area recorded every 5 days, we calculated the total intercepted photon per plant over the course of the study, as described in Legendre and van Iersel (2021).

#### 2.3.2 Photosynthetic parameters

To evaluate photosynthetic efficiency at the single-leaf level under the treatment conditions, chlorophyll fluorescence and CO_2_ exchange rate were measured on the most recently mature leaves one to three days prior to harvest. The photosynthetic measurements were conducted between 09:00 am and 5:00 pm.

Chlorophyll fluorescence measurements were carried out using a chlorophyll fluorometer (OS5p; Opti-Science, Inc., Hudson, NH, USA). To measure the minimum fluorescence (*F_o_*), the most recently mature leaves were subjected to a 30 min dark using dark adaptation clips. A saturating light pulse was then applied to determine the maximum fluorescence (*F_m_*). The maximum quantum efficiency of PSII photochemistry was calculated as *F_v_/F_m_*, where *F_v_ = F_m_ - F_o_*. To assess light-adapted photochemical efficiency under treatment conditions, maximum fluorescence (*F_m_’)* and steady-state levels of fluorescence (*F’*) were measured on light-adapted leaves. *Φ_PSII_* was calculated as (*F_m_’ - F’*)/*F_m_*’ (Baker, 2008).

Net CO_2_ assimilation rate (*P_net, light_*) and dark respiration rate (*R_dark_*) were determined using a portable gas exchange analyzer (CIRAS-3; PP systems, Amesbury, MA, USA) with the PLC3 leaf cuvette, featuring a clear top chamber (l x w; 25 mm x 18 mm). CO_2_ concentration in the cuvette was maintained at 390 μmol mol^-1^, with the cuvette air temperature set to the same as the treatment temperature (i.e., 20, 24, or 28°C). The measurements were made after leaves were placed in the leaf cuvette for 4 to 10 minutes, allowing photosynthetic rate to stabilize under the given light condition. *P_net,light_* and *R_dark_* were measured at one time point. Daily carbon gain at the single-leaf level was estimated by integrating carbon exchange rate over a 24-h period, following this equation:


$$\begin{array}{c} \text{Estimated daily carbon gain }\left({\text{mol CO}}_{2}{\text{ m}}^{-2}{\text{d}}^{-1}\right)\\ =\left(P_{net,\;light}\;\times \text{light period}-\left|R_{dark}\right|\;\times \text{dark period}\right) \end{array}$$

Where light and dark period is the length of daytime and nighttime in hour, respectively. |*R_dark_* | represents the absolute value of dark respiration rate. We assumed that the respiration rate under light and dark conditions was the same, as this is a common assumption for daily carbon gain estimations (Van Iersel, 2003; Frantz et al., 2004; Zhen and Bugbee, 2020b). Note that in the treatments with a TPFD of 150 μmol m^-2^ s^-1^ for 24 h, the dark period would be 0 hour. Photosynthetic parameters, including *Φ_PSII_* and *P_net_*, were measured under the given light treatments using incident light. A spectroradiometer (PS-100) was used to confirm that the target light conditions were achieved.

#### 2.3.3 Phytochemical analysis

For the analysis of pigments, secondary metabolites, and antioxidant capacity, the most recently mature leaves were sampled at midday, one day before harvest (23 days after treatment started). The samples were immediately immersed into liquid N_2,_ homogenized with mortar and pestle, and then stored at −80°C until further analysis.

To quantify chlorophyll and carotenoid contents, 50 mg of fresh samples were incubated in 1.5 ml of pure methanol for 24 h. Following incubation, the mixture was centrifuged at 10,000 g for 10 min to separate and collect the supernatant. The absorbance of extracts was measured at 470 nm, 652 nm, and 665 nm using a spectrophotometer (Genesys 10S ultraviolet/Vis; Thermo Fisher Scientific, Madison, WI, USA). Subsequently, chlorophylls and carotenoid contents were quantified following the method described by Wellburn (1994).

Secondary metabolites and antioxidant capacity were determined as described in Dou et al. (2019). Specifically, 100 mg of fresh samples was extracted with 0.75 ml 1% acidified methanol at 4°C in darkness. After overnight extraction, the mixture was centrifuged at 10,000 g for 10 min to obtain the supernatant for subsequent phytochemical analysis. Anthocyanin contents in the extract were quantified by measuring absorbance at 530 nm using a microplate reader (ELx800; BioTek, Winooski, VT, USA). The anthocyanin contents were expressed as milligram cyanidin-3-glucoside equivalents using a molar extinction coefficient of 29,600. For quantifying phenolic contents, the modified Folin-Ciocalteu reagent method was used: 100 μl of the extract was combined with 150 μl distilled water and 750 μl 1/10 dilution Folin-Ciocalteu reagent. After 6 min of reaction period, 600 μl 7.5 Na_2_CO_3_ solution was added to the mixture. Subsequently, the mixture was incubated at 45°C in a water bath for 10 min. The absorbance was then measured at 725 nm using the microplate reader (ELx800). The phenolic contents were quantified as milligram gallic acid equivalent per gram FW.

To determine flavonoid contents, 20 μl extract was mixed with 85 μl distilled water and 5 μl 5% NaNO_2_. After a 6-min reaction, 10 μl of 10% AlCl_3_·6H_2_O was added to the mixture. Five minutes later, 35 μl 1 M NaOH and 20 μl distilled water was added. Then, the absorbance of the mixture was measured at 520 nm using the microplate reader (ELx800). The flavonoid content was expressed as milligram of (+)-catechin hydrate equivalent per gram FW. The antioxidant capacity was assessed using the 2,2’-azino-bis (3-ethylbenzothiazoline-6-sulphonic acid) (ABTS) method by mixing 150 μl of extracts to 2.85 ml of colored free radical ABTS (ABTS^+^) solution (Arnao et al., 2001). After 10 min of reaction at room temperature, the absorbance was measured at 734 nm using the microplate reader (ELx800). The results were expressed as milligrams of Trolox equivalent antioxidant capacity per gram FW.

### 2.4 Experimental design and statistical analysis

This study was replicated two times, with four plants (subsamples) per treatment in each replicate. Vegetative growth parameters and photosynthetic efficiency were measured on all four plants in each treatment in both replicate studies. Phytochemical analysis was sampled from three plants in each treatment in the 2^nd^ replicate study. Planting density, including bordering plants, was 20.4 plants m^-2^. Treatments were arranged in a split-plot block design with temperature as the main-plot factor, photoperiod as sub-plot factor, and far-red light percentage as sub-sub-plot factor. The chamber temperature set point and the location of spectral treatments were randomized in each replicate. Subsamples were averaged before data analysis. Data were analyzed using three-way or two-way analysis of variance (ANOVA) procedure in Statistical Analysis Systems (version 9.4; SAS Inst., Inc., Cary, NC, USA). Regression analyses were performed using SigmaPlot (version 12.5; Systat Software, Inc., Chicago, IL, USA).

## 3 Results

### 3.1 Plant morphology and biomass

**Figure 1:**
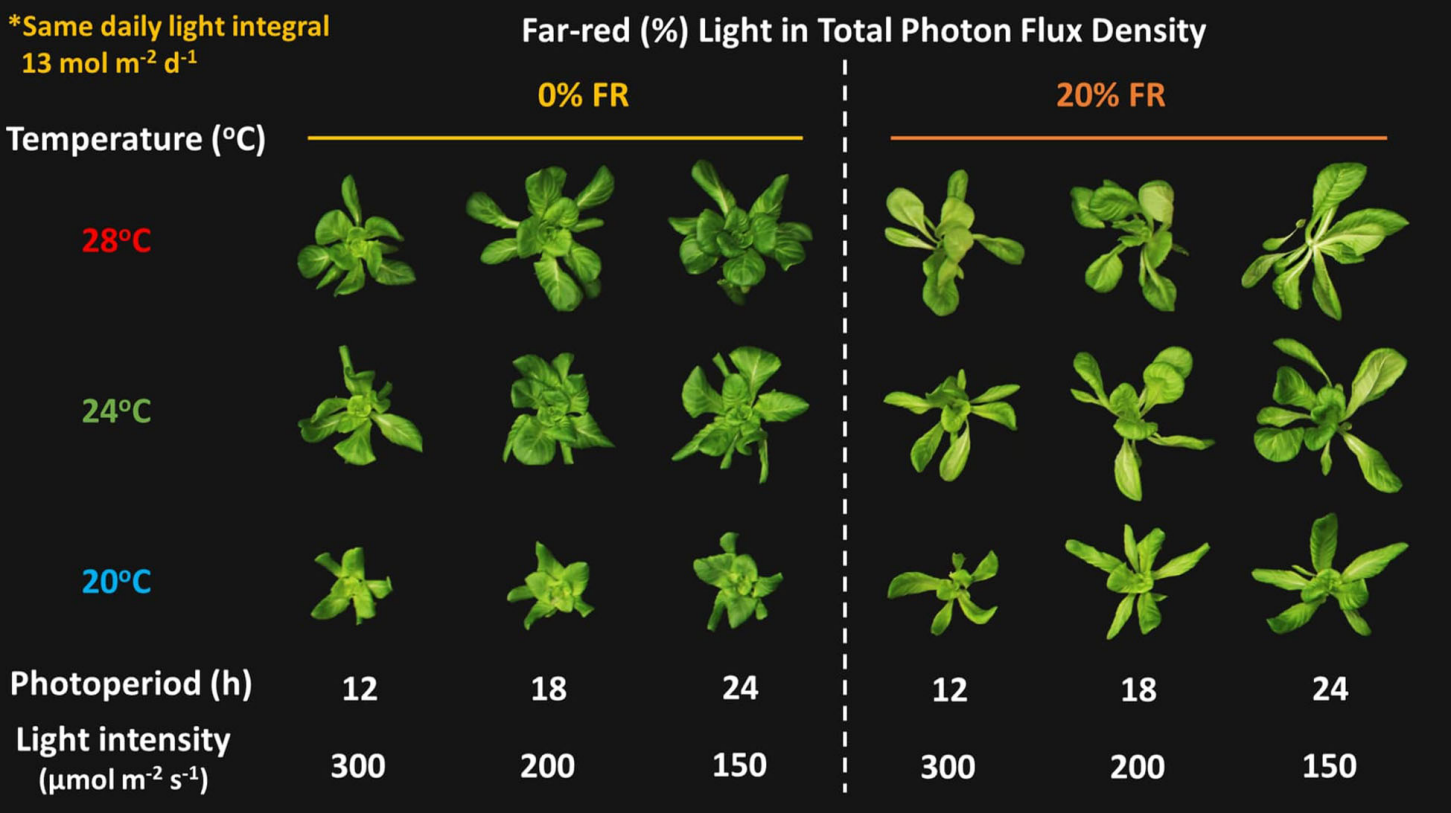
Representative lettuce plants grown under eighteen treatments composed of three temperatures (20, 24, and 28°C) x three light intensities [150, 200, and 300 μmol m^-2^ s^-1^ in total photon flux density (TPFD, 400-800 nm)] x two light spectra [0% and 20% of far-red light (FR; 700-800 nm) in TPFD]. Daily light integral was kept at 13 mol m^-2^ d^-1^ in all treatments by regulating photoperiods.

**Figure 2:**
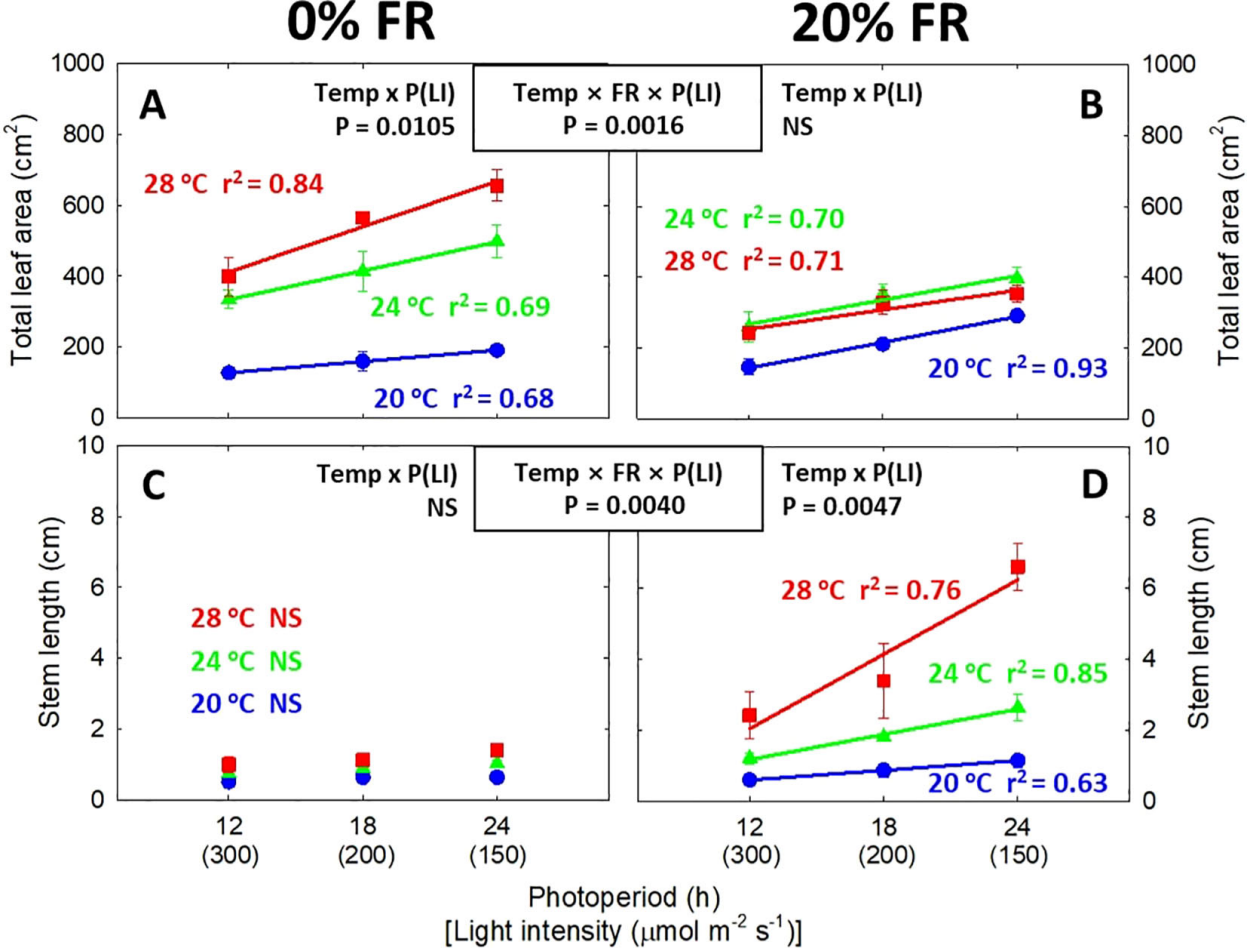
The interactive effect between light intensity [LI; 150, 200, and 300 μmol m^-2^ s^-1^ in total photon flux density (TPFD, 400-800 nm)] and temperature (Temp; 20, 24, and 28 °C) under 0% and 20% far-red light (FR; 700-800 nm) in TPFD on total leaf area **(A, B)** and stem length **(C, D)** in lettuce. To maintain the same daily light integral, longer photoperiod (P) was coupled with lower light intensity. Thus, light intensity was denoted alongside its corresponding photoperiod [i.e., photoperiod (light intensity)]. Each data point represents mean ± SE [n = 2; subsamples (4 plants per treatment per replicate study) were averaged before statistical analysis]. Coefficient of determination (r^2^) is presented when regression analysis (linear or quadratic) is statistically significant at *p*< 0.05. NS stands for non-significance.

**Figure 3:**
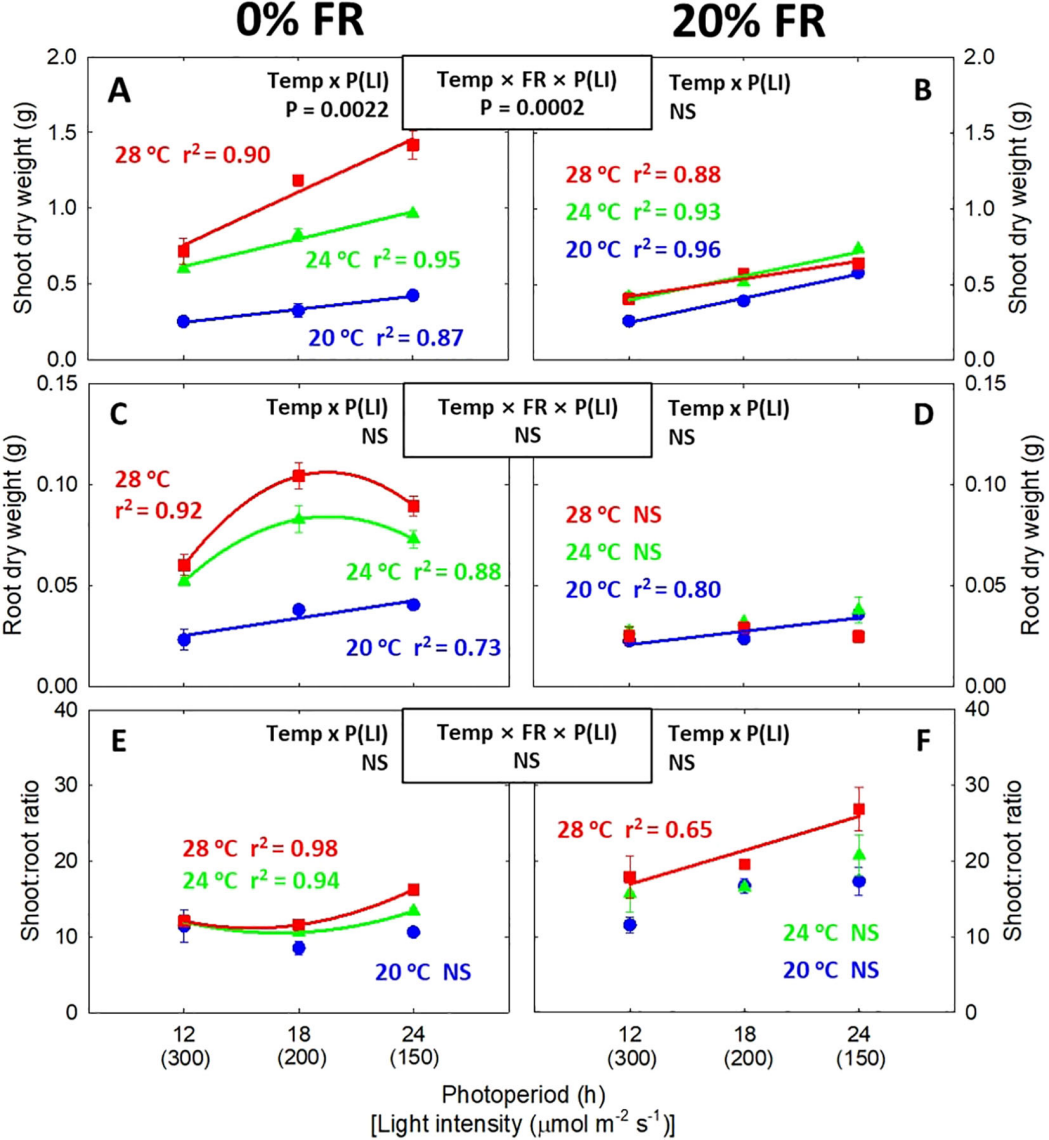
The interactive effect between light intensity [LI; 150, 200, and 300 μmol m^-2^ s^-1^ in total photon flux density (TPFD, 400-800 nm)] and temperature (Temp; 20, 24, and 28 °C) under 0% and 20% far-red light (FR; 700-800 nm) in TPFD on shoot dry weight **(A, B)**, root dry weight **(C, D)**, and shoot:root ratio **(E, F)** in lettuce. To maintain the same daily light integral, longer photoperiod (P) was coupled with lower light intensity. Thus, light intensity was denoted alongside its corresponding photoperiod [i.e., photoperiod (light intensity)]. Each data point represents mean ± SE [n = 2; subsamples (4 plants per treatment per replicate study) were averaged before statistical analysis]. Coefficient of determination (r^2^) is presented when regression analysis (linear or quadratic) is statistically significant at *p*< 0.05. NS stands for non-significance.

Significant three-way interactive effects among light intensity/photoperiod, temperature, and far-red light were observed on leaf expansion and stem elongation (**Figures 1**, **2**). Specifically, when FR light was not present, lowering light intensity from 300 to 150 μmol m^-2^ s^-1^, corresponding to extending photoperiod from 12 to 24 h, significantly promoted leaf expansion (**Figure 2A**). The effect of lowering light intensity/increasing photoperiod was more pronounced at warmer temperature, evidenced by steeper regression lines at warmer temperature, with the slope increasing from 5.3 cm^2^ h^-1^ at 20°C to 21.3 cm^2^ h^-1^ at 28°C. As a result, a significant interaction between light intensity/photoperiod and temperature was observed in total leaf area at 0% FR light (**Figure 2A**). However, at 20% FR light, there was no significant interactive effect between light intensity (or photoperiod) and temperature in leaf expansion, resulting in similar slopes of the regression lines across all three temperatures (**Figure 2B**). In contrast to leaf expansion, the interactive effect between light intensity/photoperiod and temperature on stem elongation was observed only at 20% FR light, not at 0% FR light (**Figures 2C, D**). Specifically, a lower light intensity/long photoperiod did not increase stem length at any of the three temperatures in the absence of FR light (**Figure 2C**). However, at 20% FR light, a low light intensity/long photoperiod and warm temperature synergistically stimulated stem elongation, evidenced by steeper slopes of regression lines at warmer temperature [slope (α) of 0.05 cm^2^ h^-1^ at 20°C *versus* 0.35 cm^2^ h^-1^ at 28°C] (**Figure 2D**). Shoot DW and total leaf area responded similarly to temperature, light intensity/photoperiod, and FR light treatments (**Figures 2A, B**, **3A, B**). In the absence of FR light, root DW increased when light intensity was reduced from 300 to 200 μmol m^-2^ s^-1^ (or when photoperiod was increased from 12 h to 18 h) across all three temperatures; however, further decreasing light intensity/increasing photoperiod caused a decrease in root biomass (**Figure 3C**). At 20% FR light, decreasing the light intensity or extending the photoperiod significantly increased root DW only under 20 °C at 20% FR light (**Figures 3C, D**). Shoot:root ratio increased with decreasing light intensity or increasing photoperiod under 24°C and 28 °C at 0% FR light, and under 28 °C at 20% FR light (**Figures 3E, F**). However, light intensity or photoperiod did not significantly affect specific leaf area (**Supplementary Figure 1**).

### 3.2 Photosynthetic parameters

**Figure 4:**
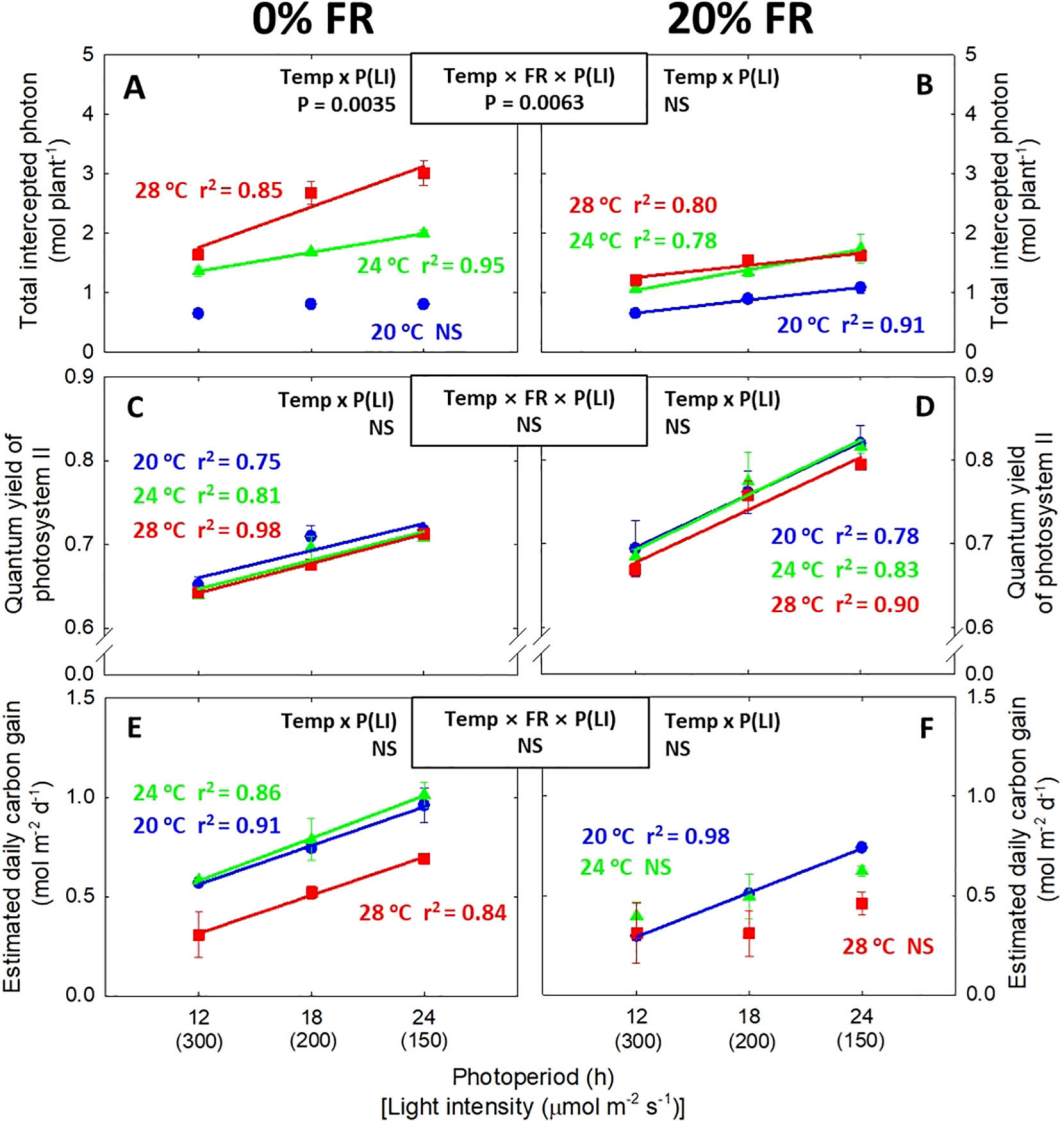
The interactive effect between light intensity [LI; 150, 200, and 300 μmol m^-2^ s^-1^ in total photon flux density (TPFD, 400-800 nm)] and temperature (Temp; 20, 24, and 28 °C) under 0% and 20% far-red light (FR; 700-800 nm) in TPFD on total intercepted photon **(A, B)**, quantum yield of photosystem II **(C, D)**, and the estimated daily carbon gain per unit leaf area **(E, F)** in lettuce. To maintain the same daily light integral, longer photoperiod (P) was coupled with lower light intensity. Thus, light intensity was denoted alongside its corresponding photoperiod [i.e., photoperiod (light intensity)]. Each data point represents mean ± SE [n = 2; subsamples (4 plants per treatment per replicate study) were averaged before statistical analysis]. Coefficient of determination (r^2^) is presented when regression analysis (linear or quadratic) is statistically significant at *p*< 0.05. NS stands for non-significance.

**Figure 5:**
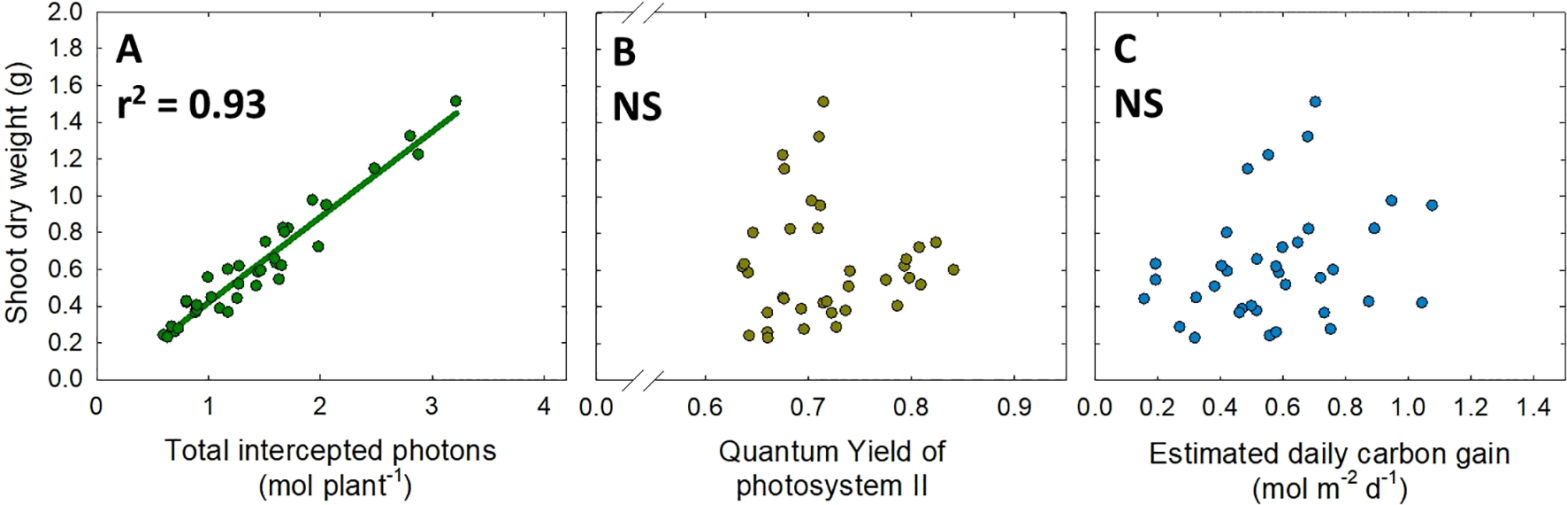
Correlation of shoot dry weight with total intercepted photons **(A)**, quantum yield of photosystem II **(B)**, and the estimated daily carbon gain per unit leaf area **(C)**. Coefficient of determination (r^2^) is presented when regression analysis (linear or quadratic) is statistically significant at *p*< 0.05. NS stands for non-significance.

Similar to total leaf area and shoot DW, total intercepted photons tended to increase in response to lower light intensity/longer photoperiod, but it was dependent on temperature and FR light level (**Figures 4A, B**). Specifically, at 0% FR light, the effect of lower light intensity/longer photoperiod was greater at warmer temperature (**Figure 4A**). In contrast, at 20% FR light, lower light intensity/longer photoperiod similarly increased total intercepted photons in all three temperatures (**Figure 4B**). Quantum yield of PSII exhibited linear increase with decreasing light intensity/increasing photoperiod (**Figures 4C, D**). For instantaneous gas exchange parameters, lower light intensity/longer photoperiod caused a decrease in *P_net_* but had no significant effect on *R_dark_* (**Supplementary Figure 2**). However, the estimated daily carbon gain increased with decreasing light intensity/increasing photoperiod (**Figures 4E, F**). Additionally, warmer temperature of 28 °C typically decreased the estimated daily carbon gain per unit leaf area (**Figures 4E, F**). In the regression analysis between plant biomass and photosynthetic parameters, total intercepted photons were highly correlated with shoot DW (r^2^ = 0.93^***^) (**Figure 5A**). However, no significant correlation was observed between shoot dry weight and single-leaf photosynthetic parameters, that is, quantum yield of photosystem II and the estimated daily carbon gain per unit leaf area (**Figures 5B, C**).

### 3.3 Pigment contents and secondary metabolites

**Figure 6:**
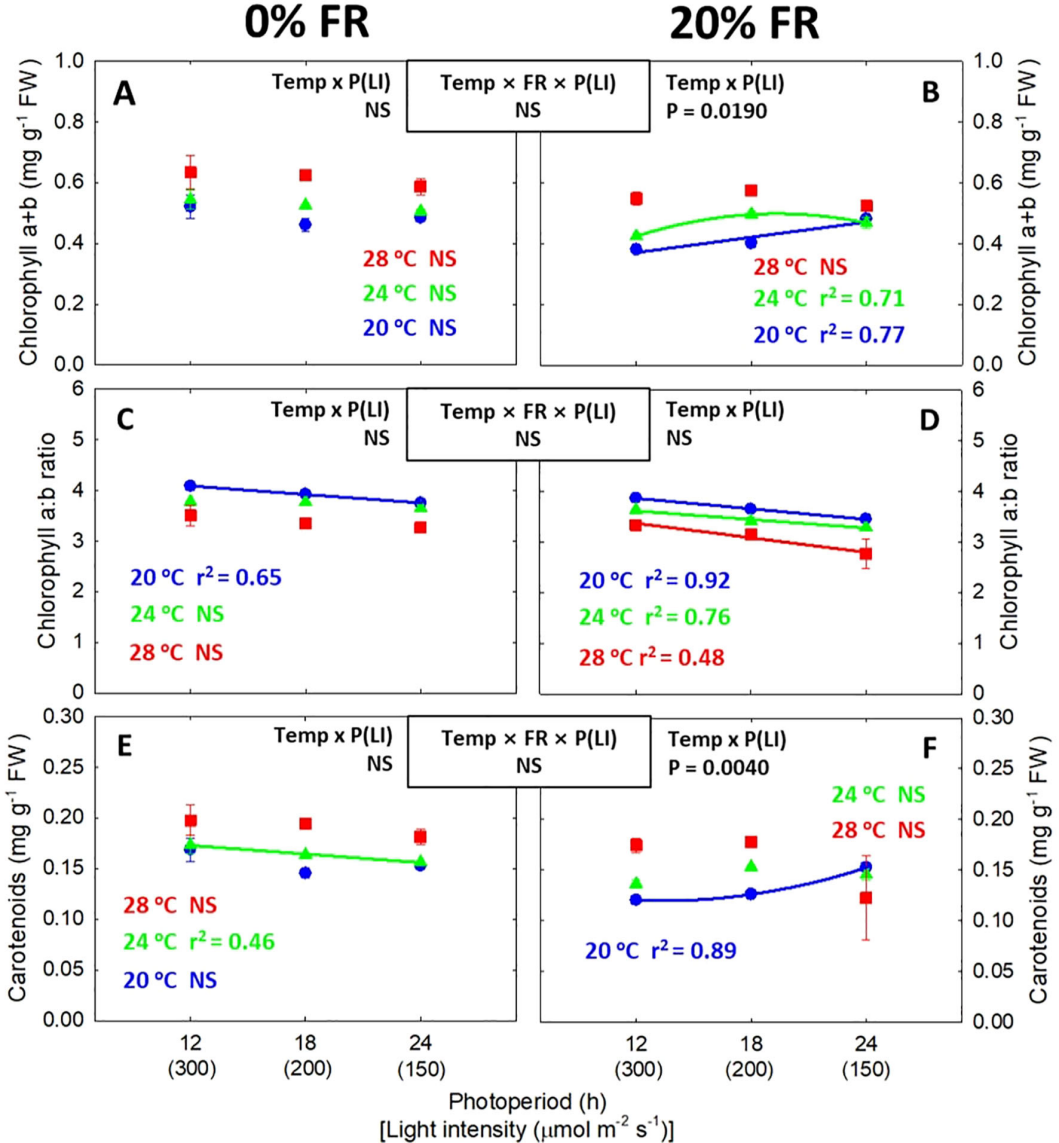
The interactive effect between light intensity [LI; 150, 200, and 300 μmol m^-2^ s^-1^ in total photon flux density (TPFD, 400-800 nm)] and temperature (Temp; 20, 24, and 28 °C) under 0% and 20% far-red light (FR; 700-800 nm) in TPFD on chlorophyll a+b content **(A, B)**, chlorophyll a:b ratio **(C, D)**, and carotenoid content **(E, F)** in lettuce. To maintain the same daily light integral, longer photoperiod (P) was coupled with lower light intensity. Thus, light intensity was denoted alongside its corresponding photoperiod [i.e., photoperiod (light intensity)]. Each data point represents mean ± SE (n = 3 from the 2^nd^ replicate study). Coefficient of determination (r^2^) is presented when regression analysis (linear or quadratic) is statistically significant at *p*< 0.05. NS stands for non-significance.

**Figure 7:**
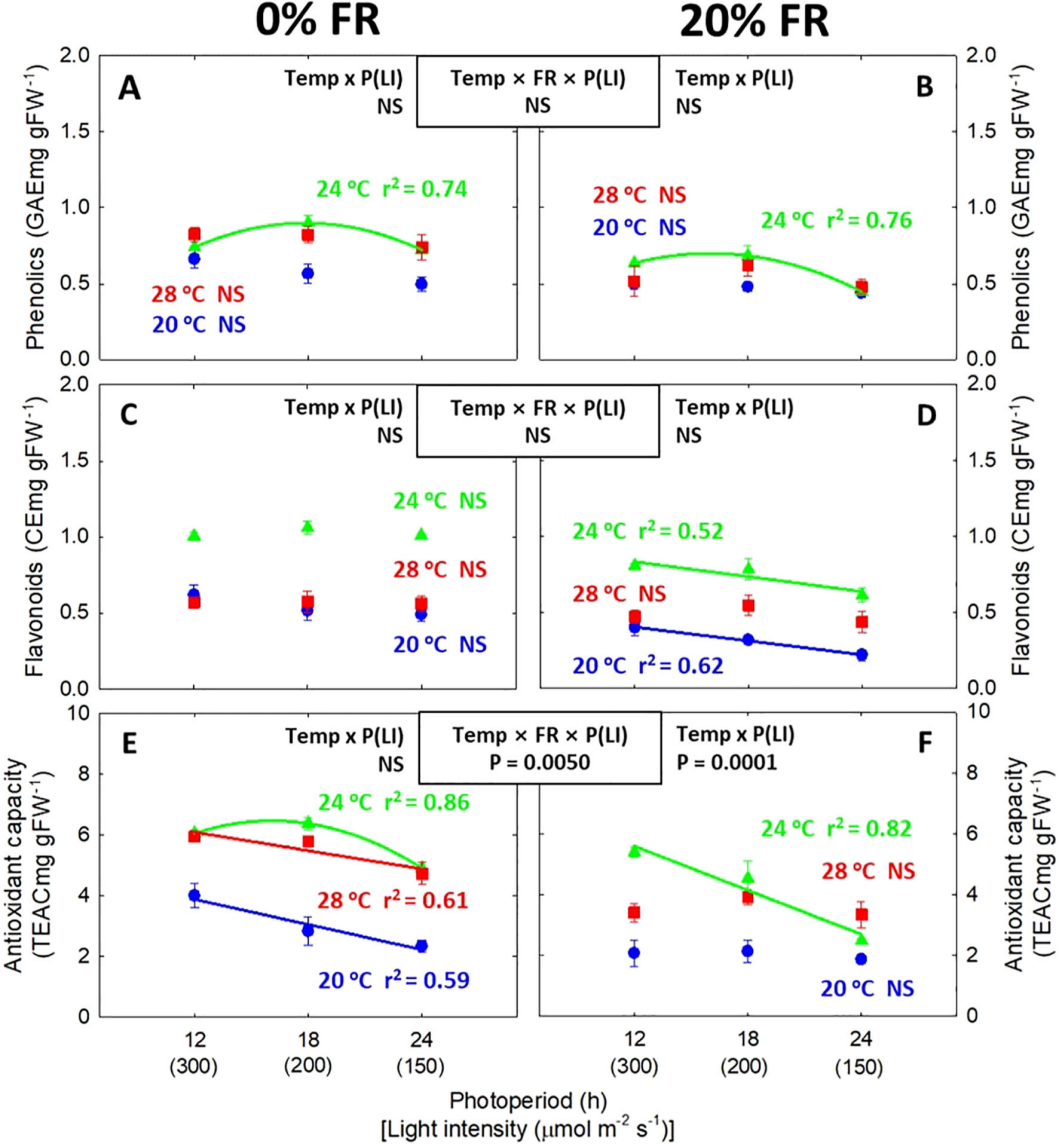
The interactive effect between light intensity [LI; 150, 200, and 300 μmol m^-2^ s^-1^ in total photon flux density (TPFD, 400-800 nm)] and temperature (Temp; 20, 24, and 28 °C) under 0% and 20% far-red light (FR; 700-800 nm) in TPFD on phenolic content **(A, B)**, flavonoid content **(C, D)**, and antioxidant capacity **(E, F)** in lettuce. To maintain the same daily light integral, longer photoperiod (P) was coupled with lower light intensity. Thus, light intensity was denoted alongside its corresponding photoperiod [i.e., photoperiod (light intensity)]. Each data point represents mean ± SE (n = 3 from the 2^nd^ replicate study). Coefficient of determination (r^2^) is presented when regression analysis (linear or quadratic) is statistically significant at *p*< 0.05. NS stands for non-significance.

Lowering the light intensity while increasing the photoperiod generally did not affect chlorophyll and carotenoid contents (**Figures 6A, B, E, F**). However, chlorophyll a:b ratio tended to decrease linearly with decreasing light intensity (**Figures 6C, D**). Similar to the photosynthetic pigments, the contents of phenolics and flavonoids were generally not sensitive to the change in light intensity (or photoperiod) (**Figures 7A–D**). However, antioxidant capacity decreased under lower light intensity/longer photoperiod conditions especially at 0% FR light (**Figures 7E, F**). Unlike a low light intensity/long photoperiod, FR light consistently decreased both pigment and secondary metabolite contents (**Supplementary Figures 3**, **4**). At the lowest light intensity (150 μmol m^-2^ s^-1^), reductions in phenolics and antioxidant capacity induced by FR light were greater under warmer temperature with statistically significant interaction (**Supplementary Figures 4C, I**).

## 4 Discussion

### 4.1 Low light intensity/long photoperiod and warm temperature synergistically enhanced leaf expansion and plant growth in the absence of FR light but predominantly stimulated stem elongation in the presence of FR light

Previous research revealed that applying a lower light intensity over a longer period, while maintaining the same DLI, effectively improved crop yield and plant productivity by enhancing leaf expansion (Soffe et al., 1977; Velez-Ramirez et al., 2014; Weaver and van Iersel, 2020; Palmer and van Iersel, 2020; Elkins and van Iersel, 2020a). In indoor farms, utilizing this lighting strategy (i.e., lower light intensity over a longer photoperiod) had further economic benefits, because growers can decrease the number of LED fixtures required for target light intensity and consequently reduce initial investment costs (Palmer and van Iersel, 2020; Warner et al., 2023). Our results are in agreement with previous research (**Figures 1**–**3**). However, the effects of a low light intensity/long photoperiod are contingent on other environmental factors, such as temperature and FR light. A particularly interesting observation was the synergistic effect between low light intensity/long photoperiod and warm temperature on leaf expansion in the absence of FR light (0% FR light), but on stem elongation in the presence of 20% FR light (**Figure 2**).

Several recent studies have reported a synergistic interaction between shade (e.g., low light intensity and high FR light) and warm temperature in various plants, including Arabidopsis, lettuce, kale, tomato, and zinnia (Romero-Montepaone et al., 2020; Burko et al., 2022; Jeong et al., 2024a, b). These studies observed a synergistic effect primarily on stem/hypocotyl elongation, suggesting enhanced shade avoidance (Legris et al., 2017; Romero-Montepaone et al., 2021). However, our research extends these findings, revealing that low light intensity and warm temperatures can also synergistically promote leaf expansion in the absence of FR light (**Figure 2A**). The synergistic effect between low light intensity/long photoperiod and warm temperature on leaf expansion provides significant practical implications for optimizing plant productivity in vertical farming systems. Specifically, lowering light intensity while increasing photoperiod at the same DLI caused a greater increase in shoot DW at 28 °C (a 96% increase), compared to 20 °C (a 68% increase), in the absence of FR light (**Figure 3A**). Consequently, the combination of low light intensity/long photoperiod and warm temperature (i.e., 150 μmol m^-2^ s^-1^/24 h x 28°C) increased shoot biomass by 463%, compared to the treatment with high light intensity/short photoperiod and cool temperature (i.e., 300 μmol m^-2^ s^-1^/12 h x 20°C) (**Figure 3A**).

However, when 20% FR light was applied, plants shifted the synergism between low light intensity/long photoperiod and warm temperature towards stem elongation, leading to a reduction in leaf expansion (**Figure 2**). The organ-specific synergism may be due to the application of FR light, which induces the transition of plant adaptative strategies in response to the combination of low light intensity, FR light, and warm temperature. Plant responses to shade signals, such as FR light and low light intensity, are generally categorized into two types: shade avoidance and shade tolerance (Gommers et al., 2013). Shade-avoiding response typically includes the elongation of hypocotyl, stem, petiole, and leaves to reach unfiltered light under vegetation shade, while shade-tolerant plants intercept more photons by expanding their leaves with a decrease in leaf thickness (Franklin, 2008; Valladares and Niinemets, 2008). Despite their different appearances, the suite of morphological adjustments is commonly interpreted as the evolutionary strategy to optimize photosynthetic carbon assimilation (carbon gain theory) (Valladares and Niinemets, 2008). Furthermore, both shade responses share a common regulatory mechanism through PHY-PIF network, where shade-tolerant traits can be facilitated by the suppression of PIFs (Molina-Contreras et al., 2019; Paulišić et al., 2021; Martinez-Garcia and Rodriguez-Concepcion, 2023). Consistent with their shared regulatory mechanism, our recent research found that plants can adopt either shade-tolerant response or shade-avoiding response depending on the level of FR light and temperature (Jeong et al., 2024b). Specifically, Jeong et al. (2024b) reported that a combination of FR light (20% of TPFD) and warm temperature (28°C) promoted stem elongation, while reducing total leaf area in six plant species, including lettuce. Thus, the excessive stem elongation at the expense of leaf expansion is likely a result of the transition from shade tolerance to shade avoidance, driven by the combined effect of FR light, low light intensity, and warm temperature. Considering the potential trade-off between stem and leaf growth, our findings underscore the importance of co-optimizing light spectra and other environmental factors when applying the synergistic leaf expansion induced by a low light intensity/long photoperiod and warm temperature to improve crop yield.

Root development is essential for plant productivity by enabling efficient water and nutrient uptake to support the growth of above-ground plant parts (Lynch, 1995; Aiken and Smucker, 1996; Fageria, 2012). However, when exposed to shade signals (i.e., lower light intensity and FR light) and warm temperature, plants tended to prioritize shoot growth over root development, increasing the shoot:root ratio (**Figures 3E, F**) (van Gelderen et al., 2018; Rosado et al., 2022). This adaptation helps plants outcompete their neighbors under unfavorable conditions for photosynthesis. In the case of lettuce, a higher shoot:root ratio is a desirable trait because shoot is the edible part of the plant. This trait is particularly beneficial in indoor farming, given that a higher harvest index is important for greater resource use efficiency.

### 4.2 The effects of environmental factors on plant biomass accumulation may depend more on photon capture, compared to single-leaf photosynthesis

The enhanced plant growth under a low light intensity/long photoperiod at a constant DLI was often attributed to the improvement of photochemical efficiency (Elkins and van Iersel, 2020b; Palmer and van Iersel, 2020). We also found a consistent increase in quantum yield of PSII with decreasing light intensity/increasing photoperiod (**Figures 4C, D**). Similar to quantum yield of PSII, the estimated daily carbon gain per unit leaf area tended to increase with lower light intensity/longer photoperiod (**Figures 4E, F**). This suggests that lower light intensity over a longer photoperiod is an advantageous strategy to improve cumulative daily photosynthesis at the single-leaf level. However, our data revealed that plant biomass did not correlate with photosynthetic parameters at the single-leaf level (i.e., quantum yield of PSII and the estimated daily carbon gain) (**Figures 5B, C**). On the other hand, the total amount of photons intercepted by the plant canopy had a much stronger correlation with shoot dry weight (r^2^ = 0.93^***^) (**Figure 5A**). The strong correlation between total intercepted photons and plant growth aligns with previous findings (Klassen et al., 2004; Elkins and van Iersel, 2020a; Kim and van Iersel, 2022). These results suggest that when applying light and temperature treatments, ensuring desirable plant morphology for photon capture is critical to maximizing plant biomass (**Figure 5A**). However, while canopy-level photon capture is essential, the efficiency of photosynthesis at single-leaf level remains critical for biomass accumulation, as the captured photons by canopy ultimately rely on the single-leaf photosynthetic efficiency. Therefore, co-optimizing both canopy structure (for maximal photon capture) and single-leaf photosynthetic efficiency is pivotal to enhancing overall plant productivity.

In this study, lowering light intensity while extending photoperiod increased canopy photon capture and the estimated daily carbon gain (**Figure 4**). Consequently, continuous light treatments (i.e., 24-hour photoperiod) produced the highest crop yield without any noticeable physiological disorders in lettuce (**Figures 1**, **3**). Sensitivity to continuous light tends to vary among species. For instance, several leafy greens, including lettuce, kale, and arugula, exhibited tolerance to continuous light (Meng and Severin, 2024). However, continuous light exposure can often induce leaf chlorosis and necrosis in some horticultural crops, such as tomato, eggplant, and geranium (Arthur et al., 1930; Murage and Masuda, 1997a; Velez-Ramirez et al., 2014). Although the underlying mechanism of species-dependent sensitivity are not fully understood, physiological disorders under continuous light have been hypothesized to result from the excessive carbohydrate accumulation in leaves and photo-oxidative damages (Velez-Ramirez et al., 2011). Furthermore, the leaf injury under continuous light can be intensified under high light intensity and warm temperature (Arthur et al., 1930; Withrow and Withrow, 1949; Murage et al., 1997b; Velez-Ramirez et al., 2011). Given these findings, further research is needed to develop species/crop-specific environmental optimization strategies to enhance crop yield without adverse physiological disorders.

### 4.3 Lowering light intensity while increasing photoperiod at a constant DLI decreased antioxidant capacity at 0% FR light, but the reduction could be compensated by warm temperature

Shade signals (i.e., FR light and low light intensity) can lead to a decrease in photosynthetic pigments (e.g., chlorophyll and carotenoids) and secondary metabolites (Lefsrud et al., 2008; Li and Kubota, 2009; Samuolienė et al., 2013; Zheng et al., 2018). This decrease may be attributed to PHY signaling, which impacts phytochemical levels in two different ways: 1) a dilution effect resulting from leaf expansion (Li and Kubota, 2009; Kong and Nemali, 2021) and 2) a direct effect on biosynthesis and degradation of both photosynthetic pigments (Casal et al., 1987; Huq et al., 2004; Toledo-Ortiz et al., 2010) and secondary metabolites (Toledo-Ortiz et al., 2010; Bianchetti et al., 2020; Pashkovskiy et al., 2022). Likewise, we observed that FR light consistently reduced the content of pigments and secondary metabolites, and antioxidant capacity (**Supplementary Figures 3**, **4**). Similarly, although the effect of a low light intensity/long photoperiod on phytochemical levels was not consistent, the lower instantaneous light intensity at the same DLI treatments significantly decreased total antioxidant capacity, regardless of temperature at 0% FR light (**Figure 7**). Moreover, FR light and warm temperature synergistically decreased phenolic contents and antioxidant capacity at lower light intensity treatments (**Supplementary Figures 4C, I**). These results suggest that the application of lower light intensity and FR light may result in a potential trade-off between crop yield and nutritional quality. However, the decreased antioxidant capacity by low light intensity and FR light can be compensated by increasing temperature (**Figures 7E, F**). Specifically, while both lower light intensity and FR light decreased antioxidant capacity by 10-50% across all the temperatures, increasing temperature from 20 to 28°C enhanced antioxidant capacity by 49-104%, regardless of light conditions (**Figures 7E, F**; **Supplementary Figures 4G–I**). The improved antioxidant capacity by warm temperature was likely derived from its impact on those secondary metabolites, considering the significant correlations of antioxidant capacity with the contents of phenolics (r^2^ = 0.70^***^) and flavonoids (r^2^ = 0.57^***^). Similar increases in various phenolics and flavonoids under warm temperatures were also reported in previous research (Lefsrud et al., 2005; Oh et al., 2009; Shamloo et al., 2017; Laddomada et al., 2021). This response may be one of the protective processes in response to heat stress (Rehman et al., 2023). However, within the temperature range (i.e., 20-28°C) in this study, severe disorder or any other visible symptoms were not observed in lettuce, supported by high values (>0.8) of F_v_/F_m_ in all temperature treatments (**Supplementary Figure 5**) (Maxwell and Johnson, 2000). Taken together, in the absence of FR light, combining lower light intensity with a longer photoperiod at the same DLI with a warm temperature (28°C) can be an effective strategy to enhance not only crop yield but also nutritional quality in terms of antioxidant capacity in lettuce production in indoor farming.

## 5 Concluding remarks

This study highlights the significant impact of the interactive effect among multiple environmental factors (i.e., FR light, light intensity/photoperiod, and temperature) on plant growth, morphology, yield and nutritional quality in indoor farming. The key finding in this study is three-way interaction between light intensity/photoperiod, warm temperature, and FR light on plant morphology. Notably, in the absence of FR light, a low light intensity/long photoperiod and warm temperature synergistically promoted leaf expansion and crop yield, without reducing secondary metabolites and antioxidant capacity. However, under 20% FR light, the synergism shifted to stem elongation, leading to a reduction in plant biomass. Thus, these results suggest that the combination of low light intensity/long photoperiod and warm temperature can serve as an effective strategy to maximize crop yield and nutrient quality in the absence of FR light.

## Data Availability

The raw data supporting the conclusions of this article will be made available by the authors, without undue reservation.
