# Supplementary material for: Lowering light intensity while extending photoperiod at a constant daily light integral synergistically interacts with warm temperature to enhance leaf expansion and crop yield in lettuce in the absence of far-red light
Source: Front Plant Sci. 2025 Jan 24;16:1529455. doi: 10.3389/fpls.2025.1529455 (PMC11803448; doi:10.3389/fpls.2025.1529455)
Supplement: Supplementary file 1 [file DataSheet1.docx]

Supplementary Material

Lowering Light Intensity While Extending Photoperiod at a Constant Daily Light Integral Synergistically Interacts with Warm Temperature to Enhance Leaf Expansion and Crop Yield in Lettuce in the Absence of Far-red Light

Sang Jun Jeong, Shuyang Zhen, Qianwen Zhang, and Genhua Niu*

* Correspondence:

Genhua Niu: [Genhua.niu@ag.tamu.edu](mailto:Genhua.niu@ag.tamu.edu)

Supplementary Figures


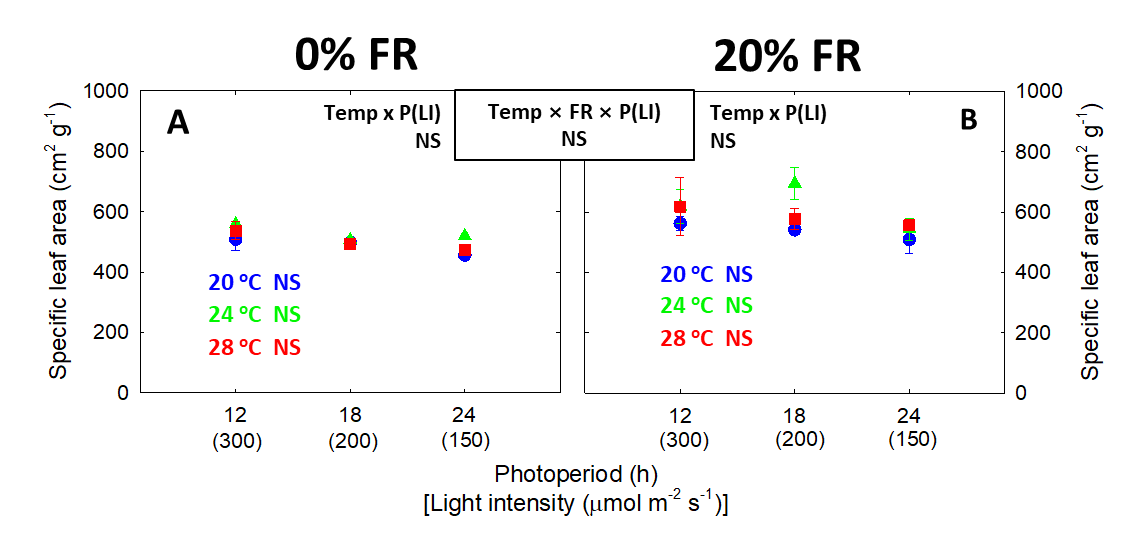


**Supplementary Figure 1.** The interactive effect between light intensity [LI; 150, 200, and 300 μmol m^-2^ s^-1^ in total photon flux density (TPFD, 400-800 nm)] and temperature (Temp; 20, 24, and 28 ℃) under 0% and 20% far-red light (FR; 700-800 nm) in TPFD on specific leaf area (A-B) in lettuce. To maintain the same daily light integral, longer photoperiod (P) was coupled with lower light intensity. Thus, light intensity was denoted alongside its corresponding photoperiod [i.e., photoperiod (light intensity)]. Each data point represents mean ± SE [n = 2; subsamples (4 plants per treatment per replicate study) were averaged before statistical analysis]. NS stands for non-significance.

**
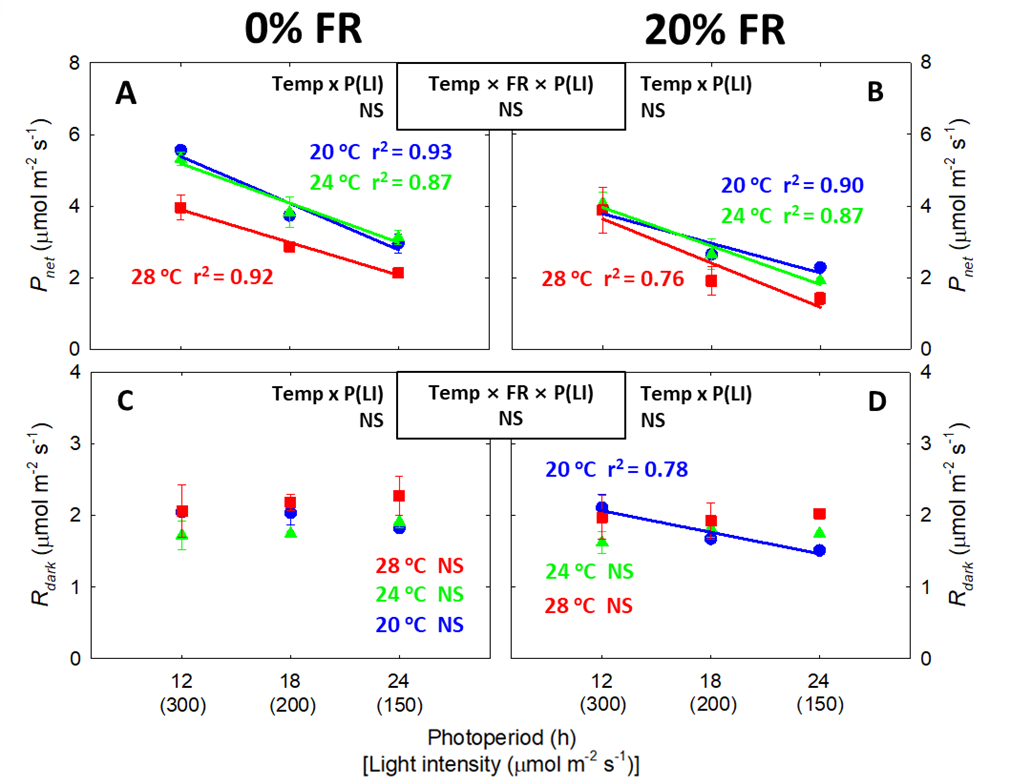
**

**Supplementary Figure 2.** The interactive effect between light intensity [LI; 150, 200, and 300 μmol m^-2^ s^-1^ in total photon flux density (TPFD, 400-800 nm)] and temperature (Temp; 20, 24, and 28 ℃) under 0% and 20% far-red light (FR; 700-800 nm) in TPFD on net CO_2_ assimilation rate (*P_net_*) (A-B) and dark respiration rate (*R_dark_*) (C-D) in lettuce. To maintain the same daily light integral, longer photoperiod (P) was coupled with lower light intensity. Thus, light intensity was denoted alongside its corresponding photoperiod [i.e., photoperiod (light intensity)]. Each data point represents mean ± SE [n = 2; subsamples (4 plants per treatment per replicate study) were averaged before statistical analysis]. Coefficient of determination (r^2^) is presented when regression analysis (linear or quadratic) is statistically significant at *p* < 0.05. NS stands for non-significance.


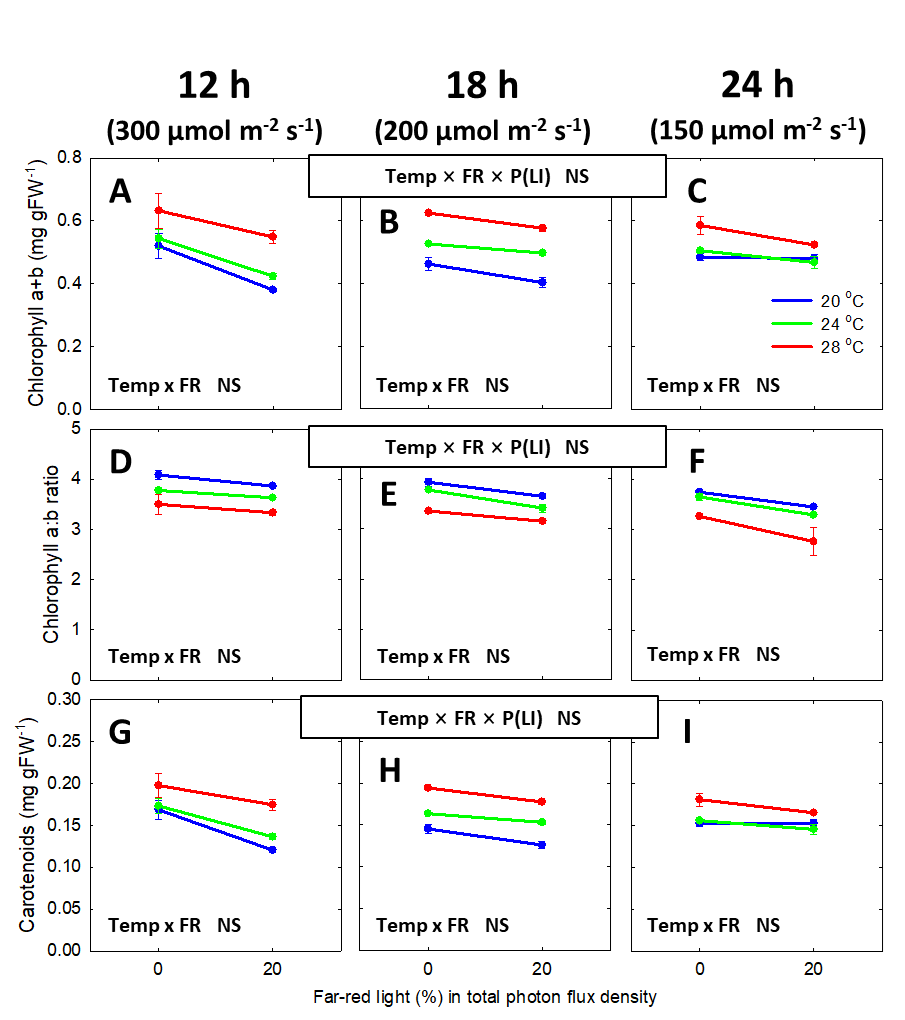


**Supplementary Figure 3.** The interactive effect between far-red light [FR (700-800 nm); 0 or 20% in total photon flux density (400-800 nm)] and temperature (Temp; 20, 24, and 28 ℃) under three different light intensities (LI; 150, 200, and 300 μmol m^-2^ s^-1^ in total photon flux density) on chlorophyll a+b content (A-C), chlorophyll a:b ratio (D-F), and carotenoid content (G-I) in lettuce. To maintain the same daily light integral, longer photoperiod (P) was coupled with lower light intensity. Thus, light intensity was denoted alongside its corresponding photoperiod [i.e., photoperiod (light intensity)]. Each data point represents mean ± SE (n = 3 from the 2^nd^ replicate study). NS stands for non-significance.


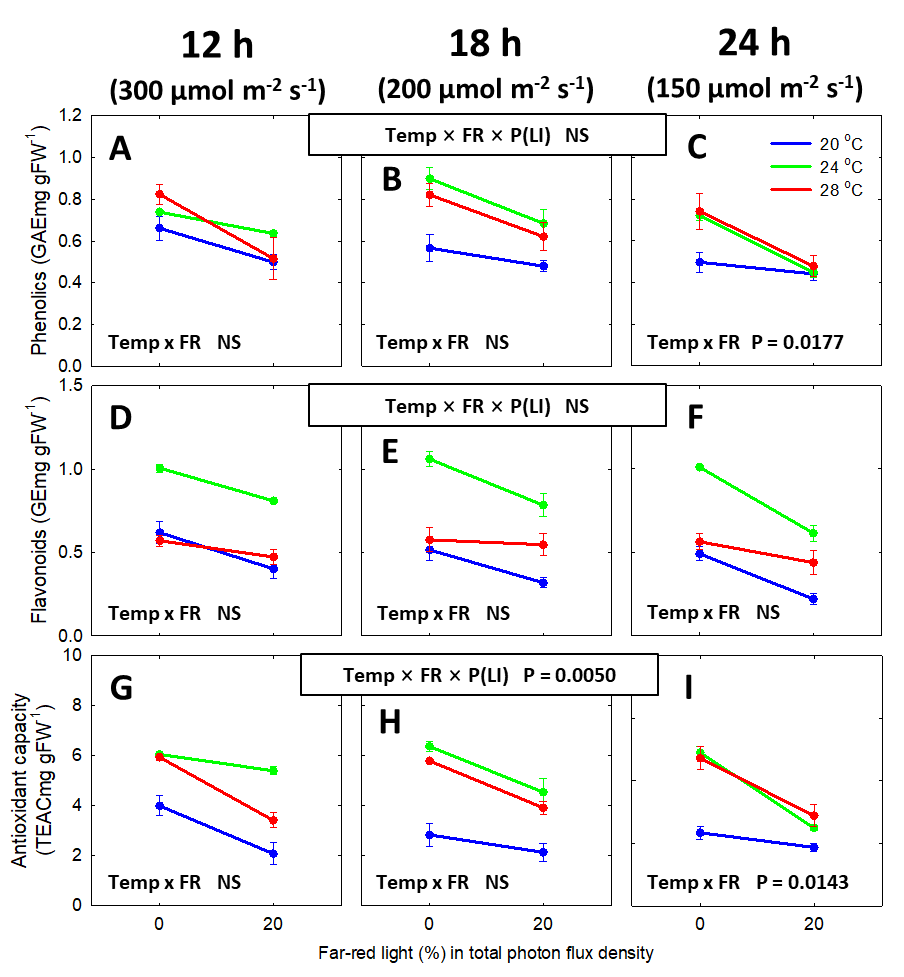


**Supplementary Figure 4.** The interactive effect between far-red light [FR (700-800 nm); 0 or 20% in total photon flux density (400-800 nm)] and temperature (Temp; 20, 24, and 28 ℃) under three different light intensities (LI; 150, 200, and 300 μmol m^-2^ s^-1^ in total photon flux density) on phenolic content (A-C), flavonoid content (D-F), and antioxidant capacity (G-I) in lettuce. To maintain the same daily light integral, longer photoperiod (P) was coupled with lower light intensity. Thus, light intensity was denoted alongside its corresponding photoperiod [i.e., photoperiod (light intensity)]. Each data point represents mean ± SE (n = 3 from the 2^nd^ replicate study). NS stands for non-significance.


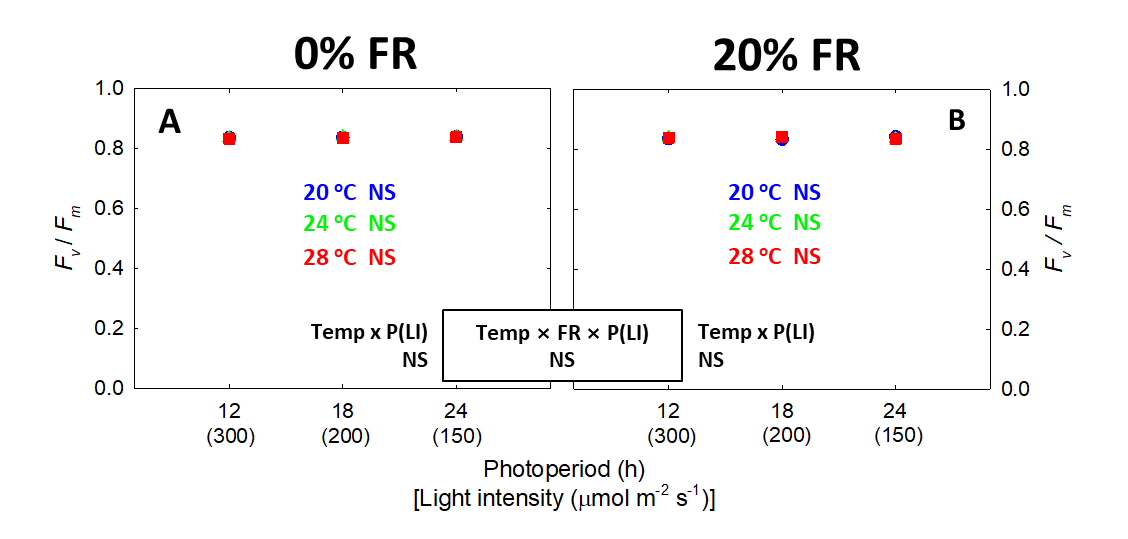


**Supplementary Figure 5.** The interactive effect between light intensity [LI; 150, 200, and 300 μmol m^-2^ s^-1^ in total photon flux density (TPFD, 400-800 nm)] and temperature (Temp; 20, 24, and 28 ℃) under 0% and 20% far-red light (FR; 700-800 nm) in TPFD on the maximum quantum efficiency of PSII photochemistry (*F_v_/F_m_*) (A-B) in lettuce. To maintain the same daily light integral, longer photoperiod (P) was coupled with lower light intensity. Thus, light intensity was denoted alongside its corresponding photoperiod [i.e., photoperiod (light intensity)]. Each data point represents mean ± SE [n = 2; subsamples (4 plants per treatment per replicate study) were averaged before statistical analysis]. NS stands for non-significance.
